# Age-Based Disparities in Metastatic Melanoma Patients Treated in the Immune Checkpoint Inhibitors (ICI) *Versus* Non-ICI Era: A Population-Based Study

**DOI:** 10.3389/fimmu.2021.609728

**Published:** 2021-11-16

**Authors:** Mohammed Safi, Mahmoud Al-Azab, Chenxing Jin, Dario Trapani, Salem Baldi, Salah Adlat, Aman Wang, Bashir Ahmad, Hamza Al-madani, Xiu Shan, Jiwei Liu

**Affiliations:** ^1^ Department of Oncology, First Affiliated Hospital of Dalian Medical University, Dalian, China; ^2^ Department of Immunology, Guangzhou Women and Children’s Medical Centre, Guangzhou Medical University, Guangzhou, China; ^3^ IEO - Istituto Europeo di Oncologia, Milan, Italy; ^4^ Department of Clinical Biochemistry, College of Laboratory Diagnostic Medicine, Dalian Medical University, Dalian, China; ^5^ Guangdong Cardiovascular Institute, Guangdong Provincial People’s Hospital, Guangzhou, China; ^6^ Department of Biology, The University of Haripur, Haripur, Pakistan; ^7^ Cixi Institute of Biomedical Engineering, Ningbo Institute of Materials Technology and Engineering, University of Chinese Academy of Sciences, Ningbo, China

**Keywords:** age distribution, melanoma, SEER database, epidemiology, immune-checkpoint inhibitor (ICI)

## Abstract

Immune checkpoint inhibitors (ICIs) have revolutionized metastatic melanoma treatment, but our knowledge of ICI activity across age groups is insufficient. Patients in different age groups with advanced melanoma were selected based on the ICI approval time in this study. Patients with melanoma were identified in the Surveillance, Epidemiology, and End Result (SEER) database program 2004–2016. The results showed that 4,040 patients had advanced melanoma before the advent of ICI (referred to as the “non-ICI era”), whereas there were 6,188 cases after ICI approval (referred to as the “ICI era”). In all age groups, the cases were dominated by men. The differences between the first (20–59 years) and second (60–74 years) age groups in both eras were significant in terms of surgery performance and holding of insurance policies (*p* = 0.05). The first and second groups (20–59 and 60–70 years old, respectively) showed no difference in survival (median = 8 months) during the non-ICI era, but the difference was evident in the first, second, and third age groups in the ICI era, with the younger group (20–59 years) having significantly better survival (median = 18, 14, and 10 months, respectively, *p* = 0.0001). Multivariate analysis of the first group (the youngest) in the ICI era revealed that surgery was significantly associated with an increase in survival among patients compared with those who did not undergo surgery (*p* < 0.0001). Furthermore, having an insurance policy among all age groups in the ICI era was associated with favorable survival in the first (20–59 years) and second (60–74 years) age groups (*p* = 0.0001), while there were no survival differences in the older ICI group (>74 years). Although there were differences in survival between the ICI era and the non-ICI era, these results demonstrate that ICI positively affected the survival of younger patients with advanced melanoma (first age group) than it had beneficial effects on older patients. Moreover, having had cancer surgery and holding an insurance policy were positive predictors for patient survival. This study emphasizes that adequate clinical and preclinical studies are important to enhance ICI outcomes across age groups.

## Introduction

The first immune check inhibitor (ICI) was approved for melanoma treatment in 2011 (ipilimumab). Immunotherapy has demonstrated a significant improvement in progression-free and overall survival (OS) compared with other therapeutic approaches across multiple cancers and has revolutionized the therapeutic landscape of melanoma (1, 2). Cytotoxic T-lymphocyte-associated protein-4 (CTLA-4) and programmed death protein-1 (PD-1) have emerged as the standard ICIs, establishing a valuable role in the curative and non-curative settings (3).

Aging is associated with increased immune dysfunction involving notable changes in the innate and adaptive immunity (4). Hematopoiesis is most strongly biased toward myeloid development, whereas lymphopoiesis retracts with age. Various age-related changes common to peripheral T-cell populations include less naive T cells, high numbers of terminally differentiated T cells, and reduced expressions of co-stimulatory molecules. Although recent studies have reported variations in the age-related outcomes in different cancer types (5–7), they are mostly short reports and case series and did not discuss the specific age groups with their clinical traits in a large cohort. In the setting of elderly patients with melanoma, there is an unmet systemic need; the evidence is limited to the correlative analysis between age ranges and treatment response, and no population-based study has been conducted (8).

In this work, we addressed the research gap in the efficacy of ICI patients with melanoma by evaluating the effects of ICI on a large cohort of patients with advanced melanoma and analyzing the association with OS in specific age groups.

## Study Cohorts

The ethical statement of permission to access the SEER research data files was obtained by using SEER 18 Regs Custom Data (with additional treatment fields), Nov 2018 Sub (1975–2016 varying). According to the exemption regulations, use of the data released by the SEER database does not require informed patient consent. Patients were identified from SEER 2018 with additional treatment fields through SEER*Stat software (version 8.3.9.1). The SEER program of the National Cancer Institute is responsible for the collection and reporting of cancer incidence and survival data from several populations based on central cancer registries that cover approximately 30% of the US population. The SEER data include patient demographic information, primary tumor site, tumor morphology, stage at diagnosis, the first course of cancer treatment, and vital status.

**Figure 1:**
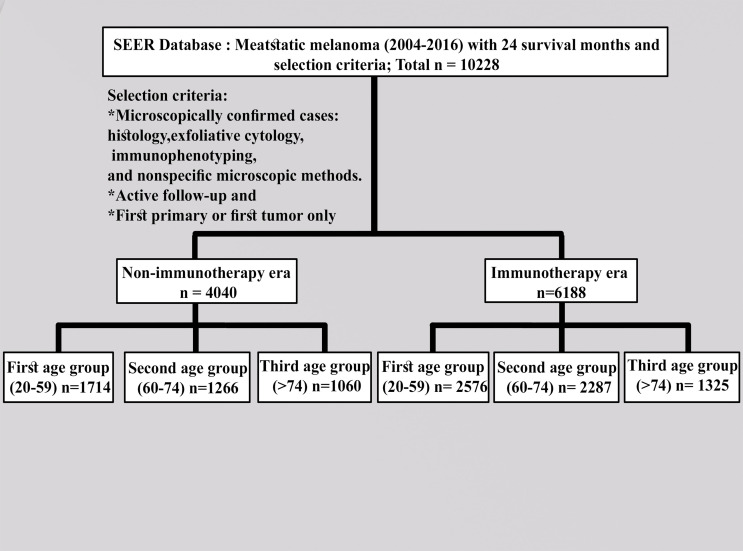
Detailed description of the inclusion criteria.

We collected the SEER data for the cohort of patients from the latest registry with additional treatment fields using the SEER*Stat software (version 8.3.9.1). Following the US Food and Drug Administration approval of ICI use (9), the appropriate codes for advanced melanoma (III and IV) were selected as site recode ICD-3/WHO2008 (melanoma of the skin) according to the 6th AJCC edition (2004–2015) and Derived SEER Cmb Stg Grp (2016 onwards). Furthermore, 2004–2010 was selected and compared with the 2011–2016 period to investigate the effects of this variable on patients with advanced melanoma. All patients were designated based on follow-up (active follow-up). Only those microscopically confirmed cases *via* histology, exfoliative cytology, immunophenotyping, and nonspecific microscopic methods, active follow-up, and first primary or first only were included. The following variables were evaluated: age groups (20–59 years as the first group, 60–74 years as the second group, and >74 years as the third group); year of diagnosis by contemporary intervals; all months of survival; grade (I–II, III–IV, or other); sex (male or female); available information (2011–2016) on the sites of metastasis (mets) (bone, brain, liver, or lung); primary site labeled (trunk, upper limb, lower limb, or others); race (white, black, or other); radiation therapy (beam radiation or radioisotopes); radioactive implants (yes or no); chemotherapy (yes or no); vital status (dead or alive); laterality (right, left, or others); patient ID; marital status; and holder of an insurance policy or not. Known survival with 24 months as the cutoff value was selected. The detailed criteria for inclusion and exclusion are described in **Figure 1**.

Patients’ baseline demographics were compared using *χ* ^2^ test depending on the diagnosis of advanced melanoma. The median OS was analyzed using the Kaplan–Meier method *via* a log-rank test, and the Cox proportional hazard model was used for multivariate analysis in SPSS. Statistical significance was considered at *p*-values less than 0.05 and a limit of 0.0001.

## Results

We extracted 10,228 cases with advanced melanoma, 4,040 of which were recorded before the advent of ICI (non-ICI era) and 6,188 cases had the same disease when ICI was developed (ICI era), with 24 months as the survival cutoff. The cases predominantly consisted of males. Results of the comparison of both eras for the first and second groups revealed that laterality; chemotherapy, radiation therapy, and surgery; and the holding of an insurance policy were significantly different in the first and second age groups (*p* ≤ 0.05), whereas only radiation therapy and surgery were not in the third age group (*p* = 0.0001). These characteristics are summarized in **Table 1**.

**Table 1:** Characteristics of advanced melanoma patients.

Parameters	20–59 years		*p*-value	60–74 years		*p*-value	>74 years		*p*-value
	Non-ICI era: *n* = 1,714 (17%)	ICI era: *n* = 2,576 (25%)		Non-ICI era: *n* = 1,266 (12%)	ICI era: *n* = 2,287 (22%)		Non-ICI era: *n* = 1,060 (11%)	ICI era: *n* = 1,325 (13%)	
**Sex**									
**Male**	1,193 (69.6)	1,621 (62.9)	0.0001	899 (71.0)	1,621 (70.9)	0.0001	662 (62.5)	837 (63.2)	0.733
**Female**	521 (30.4)	955 (37.1)		367 (29.0)	666 (29.1)	398 (37.5)	488 (36.8)
**Race**									
**White**	1,649 (96.5)	2,468 (96.2)	0.689	1,207 (95.3)	2,191 (96.4)	0.219	1,014 (95.8)	1,269 (96.5)	0.609
**Black**	27 (1.6)	40 (1.6)		28 (2.2)	33 (1.5)	21 (2.0)	20 (1.5)
**Others**	32 (1.9)	58 (2.3)		31 (2.4)	50 (2.2)	24 (2.3)	26 (2.0)
**Marital status**									
**Yes**	906 (52.9)	1,343 (52.1)	0.662	779 (61.5)	1,342 (58.7)	0.101	531 (50.1)	688 (51.9)	0.387
**Others**	808 (47.1)	1,233 (47.9)		487 (38.5)	945 (41.3)	529 (49.9)	637 (48.1)
**Laterality**									
**Right**	395 (23.0)	823 (31.9)	0.0001	264 (20.9)	636 (27.8	0.0001	264 (24.9)	343 (25.9)	0.030
**Left**	443 (25.8)	786 (30.5)		269 (21.2)	650 (28.4)	236 (22.3)	348 (26.3)
**Others**	876 (51.1)	967 (37.5)		733 (57.9)	1,001 (43.8)	560 (52.8)	634 (47.8)
**Primary site labeled**									
**Trunk**	446 (26)	695 (27)	0.0001	236 (18.6)	566 (24.7)	0.0001	204 (15.4)	168 (15.8)	0.948
**Upper site**	175 (10.2)	316 (12.3)		123 (9.7)	299 (13.1)	183 (13.8)	138 (13)
**Lower site**	217 (12.7)	494 (19.2)		150 (11.8)	321 (14)	204 (15.4)	164 (15.5)
**Others**	876 (51.1)	1,071 (41.6)		757 (59.8)	1,101 (48.1)	734 (55.4)	590 (55.7)
**Surgery status**									
**Performed**	913 (53.6)	1,683 (65.4)	0.0001	621 (49.2)	1,374 (60.3)	0.0001	549 (52.3)	726 (54.9)	0.214
**Others**	791 (46.4)	891 (34.6)		641 (50.8)	906 (39.7)	501 (47.7)	597 (45.1)
**Radiation status**									
**Yes**	598 (35.3)	609 (23.8)	0.0001	407 (32.4)	551 (24.3)	0.0001	231 (21.9)	252 (19.2)	0.112
**No**	1,098 (64.7)	1,946 (76.2)		851 (67.6)	1,721 (75.7)	824 (78.1)	1,063 (80.8)
**Chemotherapy**									
**Yes**	596 (34.8)	513 (19.9)	0.0001	382 (30.2)	363 (15.9)	0.0001	141 (13.3)	135 (10.2)	0.020
**No**	1,118 (65.2)	2,063 (80.1)		884 (69.8)	1,924 (84.1)	919 (86.7)	1,190 (89.8)
**Metastasis site**									
**Bone**									
**Yes**		265 (10.5)			273 (12.3)			134 (10.5)	
**No**		2,251 (89.5)			1,943 (87.7)			1,147 (89.5)	
**Brain**									
**Yes**		444 (17.6)			434 (19.4)			210 (16.4)	
**No**		2,077 (82.4)			1,804 (80.6)			1,070 (83.6)	
**Liver**									
**Yes**		292 (11.6)			314 (14.1)			175 (13.6)	
**No**		2225 (88.4)			1,905 (85.9)			1,109 (86.4)	
**Lung**									
**Yes**		477 (19)			541 (24.3)			360 (28.0)	
**No**		2,035 (81)			1,684 (75.7)			928 (72)	
**Insurance**									
**Yes**	845 (49.30)	2,347 (91.1)	0.0001	725 (57.27)	2,164 (94.6)	0.0001	596 (56.23)	1,266 (95.5)	0.0001
**Others**	154 (8.98)	229 (8.9)		46 (3.63)	123 (5.4)	31 (2.92)	59 (4.5)
**Missed**	715 (41.72)	0 (0)		495 (39.1)	0 (0)	433 (40.85)	0 (0)

ICI, immune checkpoint inhibitor.

**Figure 2:**
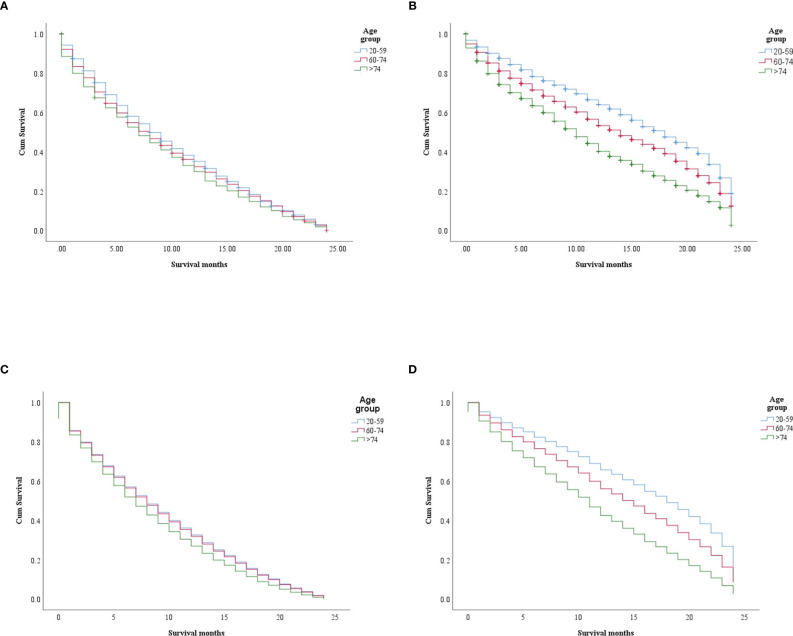
Survival differences in the age groups within each era (non-ICI and ICI). **(A, B)** Kaplan–Meier (KM) survival difference in the non-ICI and the ICI era (*p* = 0.0004 and *p* < 0.0001, respectively). **(C, D)** Multivariate Cox models for the age groups in the non-ICI era (20–59 years: *p* = 0.0001; 60–74 years: HR = 1.01, CI = 0.94–1.09, *p* = 0.614; >74 years : HR = 1.16, CI = 1.07–1.26, *p* = 0.0001) **(C)** and the ICI era (20–59 years: *p* < 0.0001; 60–74 years: HR = 1.37, CI = 1.261.50, *p* < 0.0001; >74 years: HR = 2.04, CI = 1.85–2.24, *p* < 0.0001) **(D)**. *ICI*, immune checkpoint inhibitor.

**Figure 3:**
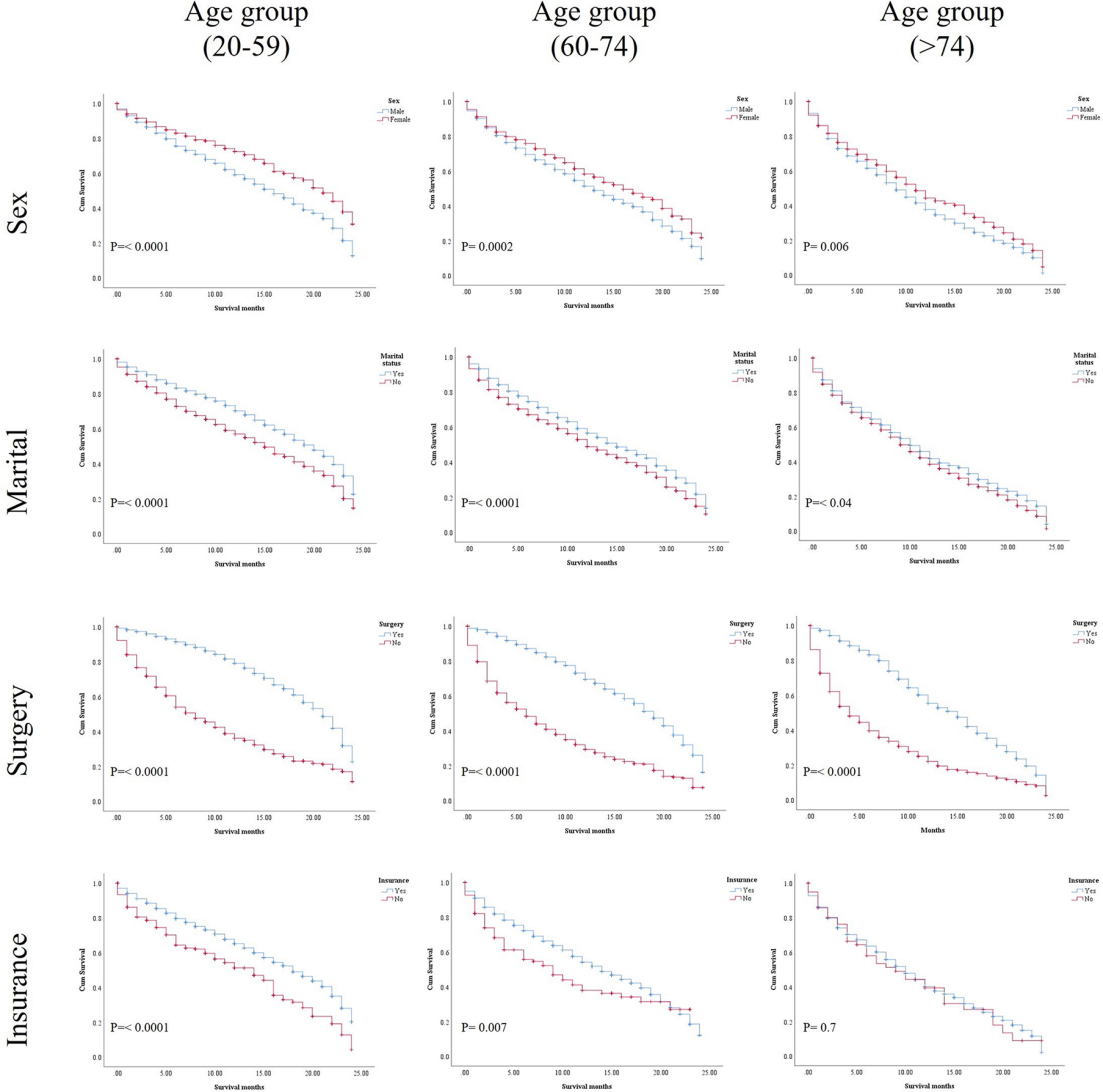
Kaplan–Meier (KM) results of the age groups in the ICI era for the selected variables: sex, marital status, surgery status, and insurance status.

**Figure 4:**
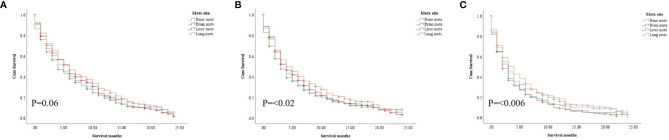
Survival pattern of the presence of metastatic sites in each age group of the ICI era. **(A)** First age group, 20–59 years. **(B)** Second age group, 60–74 years. **(C)** Third age group, >74 years.

**Figure 5:**
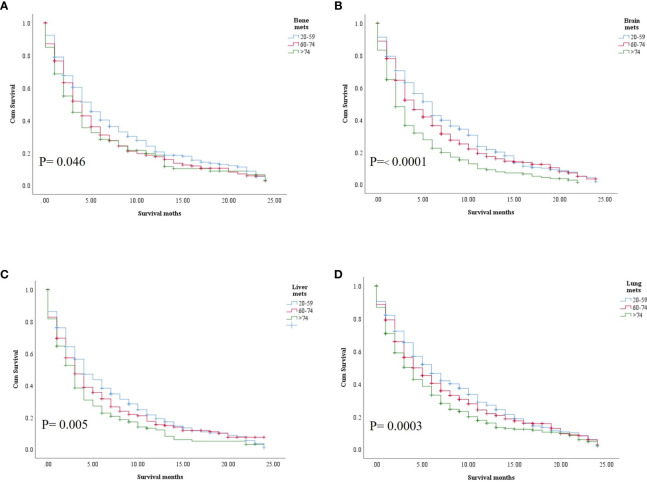
Comparative sites of metastasis among the age groups. **(A)** Bone. **(B)** Brain. **(C)** Liver. **(D)** Lung.

In the study of age group disparities in OS differences between the non-ICI and the ICI era, it was revealed that there were no significant survival differences in the first and second groups in the non-ICI era (median = 8 months) and that the older group had worse survival [median = 7 months, hazard ratio (HR) = 1.16, CI = 1.07–1.26, *p* = 0.0001], while the difference between the groups in the ICI era was clearly shown in the first, second, and third age groups (median = 18, 14, and 10 months, respectively, *p* < 0.0001) (**Figure 2**). We studied the general OS difference between the non-ICI and ICI eras, with significant positive survival for those in the ICI era (*p* < 0.0001) (**Supplementary File 1**). We considered three age groups to classify the patients. Across the age cohorts, we found differences in OS only in the first group in the comparison of patients treated with or without ICI (i.e., in the pre-ICI or the ICI era), with an improvement in the median survival of almost all variables, sex, marital status, surgery use, and insurance, in the ICI era (*p* < 0.05) (**Table 2**). By studying differences in OS in the ICI group, we found that being female, married, and having had surgery had beneficial favorable survival (*p* = 0.05). Whereas having insurance had better survival for the first (20–59 years) and second (60–74 years) groups (*p* = 0.05), there was no difference in insurance use for the old group (*p* = 0.7) (**Figure 3**). In the study of the sites of metastasis and their effects on survival in the age groups of the ICI era, we found that mets sites have worse survival than do non-metastatic cases (**Supplementary File 2**). In the comparative study of the sites of metastasis in the age groups of the ICI era, a difference was shown and was associated with poor survival in the old group in cases of brain, liver, and lung metastases (*p* = 0.05). Bone metastasis showed fair association and only significant marginal differences were presented in the first age group (*p* = 0.046) (**Figure 4**). In the study of the differences in the mets sites in the ICI group, liver metastasis was associated with shorter median months of survival in the first (20–59 years) and second (60–74 years) age groups (median = 4 *vs*. 3 months), whereas brain metastasis was associated with worse survival in the older group (>74 years; median = 2 months, *p* = 0.006) (**Figure 5**).

**Table 2:** Survival patterns of the age groups in the non-immunotherapy and the immunotherapy era.

Parameters	Median (months)			Log rank		Median (months)				Log rank		Median (months)					Log rank
	Non-ICI era: 20–59 years	ICI era: 20–59 years				Non-ICI era: 60–74 years		ICI era: 60–74 years				Non-ICI era: >74 years		ICI era: >74 years			
**Sex**																	
**Male**	8.00	16.00		0.000		7.00		13.00		0.000		7.00		9.00			0.000
**Female**	9.00	21.00		0.000		8.00		16.00		0.000		7.00		11.00			0.000
**Race**																	
**White**	8.00	18.00		0.000		7.00		14.00		0.000		7.00		10.00			0.000
**Black**	12.00	14.00		0.059		12.00		13.00		0.177		10.00		11.00			0.429
**Others**	11.00	20.00		0.000		12.00		15.00		0.042		12.00		11.00			0.991
**Marital status**																	
**Yes**	9.00	20.00		0.000		8.00		15.00		0.000		8.00		10.00			0.000
**Others**	7.00	15.00		0.000		7.00		12.00		0.000		6.00		9.00			0.000
**Laterality**																	
**Right**	12.00	21.00		0.000		12.00		18.00		0.000		11.00		13.00			0.000
**Left**	12.00	21.00		0.000		11.00		18.00		0.000		12.00		13.00			0.000
**Others**	6.00	10.00		0.000		5.00		8.00		0.000		4.00		5.00			0.000
**Primary site**																	
**Trunk**	10.00	21.00		0.000		10.00		16.00		0.000		9.00		11.00			0.001
**Upper site**	13.00	21.00		0.000		11.00		18.00		0.000		12.00		14.00			0.001
**Lower site**	12.00	22.00		0.000		12.00		19.00		0.000		12.00		16.00			0.001
**Others**	6.00	11.00		0.000		5.00		10.00		0.000		4.00		7.00			0.000
**Surgery**																	
**Performed**	13.00	21.00		0.000		12.00		19.00		0.000		11.00		15.00			0.000
**Others**	5.00	8.00		0.000		4.00		6.00		0.000		3.00		4.00			0.000
**Radiation status**																	
**Yes**	6.00	10.00		0.000		5.00		7.00		0.000		5.00		6.00			0.015
**No**	10.00	21.00		0.000		9.00		17.00		0.000		8.00		11.00			0.000
**Chemotherapy**																	
**Yes**	8.00	11.00		0.000		8.00		9.00		0.000		7.00		8.00			0.486
**No**	9.00	20.00		0.000		7.00		16.00		0.000		7.00		10.00			0.000
**Insurance**																	
**Yes**	9.00	18.00		0.000		8.00		14.00		0.000		8.00		10.00			0.000
**No**	7.00	14.00		0.000		6.00		9.00		0.025		5.00		9.00			0.052

ICI, immune checkpoint inhibitor.

In the univariate analysis, being female, married, having had surgery, and insured patients showed significant positive survival benefits in the three groups of the ICI era. The multivariate analysis of each group revealed that, in the first (20–59 years) and second (60–74 years) age groups in the ICI era, being female, married, insured, and had surgery performed were significantly associated with an increase in survival compared with those who did not undergo surgery (HR = 1.536, CI = 1.258–1.874, *p* < 0.0001; HR = 2.103, CI = 1.750–2.527, *p* = 0.0001), whereas only surgery was an independent factor, where having undergone surgery was related with better survival than non-surgery in the older group. The presence of metastatic sites was associated with lower survival in all age groups (*p* = 0.0001) and increased significant OS difference between mets in the same group with increasing age (**Tables 3A–C**).

Table 3 AUnivariate and multivariate analysis of factors affecting the survival in non-ICI era and ICI in first age group.

**Table:** 

Parameters	( 20-59 years) Non-ICI era				( 20-59 years) ICI era			
	Univariate HR (CI)	P value	Multivariate HR (CI)	P value	Univariate HR (CI)	P value	Multivariate HR (CI)	P-value
Sex Male vs. female	0.937(0.844-1.041)	0.228			0.656(0.575-0.748)	0.000	0.736(0.640-0.846)	0.000
Race								
White		0.996				0.523		
Black	0.984(0.673-1.440)	0.936			1.290(0.829-2.009)	0.259		
Others	1.004(0.708-1.425)	0.981			0.975(0.656-1.450)	0.902		
Marital	1.144(1.039-1.260)	0.006	1.209(1.095-1.334)	0.000	1.501(1.332-1.692)	0.000	1.464(1.290-1.661)	0.000
Primary site								
Trunk		0.000		0.391		0.000		0.505
Upper site	0.928(0.776-1.110)	0.415	0.902(0.751-1.084)	0.271	0.950(0.756-1.195)	0.663	0.965(0.761-1.225)	0.772
Lower site	0.870(0.737-1.028)	0.102	0.868(0.732-1.030)	0.106	0.810(0.654-1.003)	0.053	0.847(0.675-1.062)	0.150
Others	1.562(1.390-1.756)	0.000	0.959(0.801-1.148)	0.651	2.206(1.897-2.566)	0.000	0.913(0.730-1.141)	0.423
Laterality								
Right		0.000		0.815		0.000		0.123
Left	1.015(0.884-1.167)	0.830	1.046(0.909-1.205)	0.529	0.939(0.790-1.117)	0.480	1.002(0.836-1.199)	0.987
Others	1.635(1.448-1.847)	0.000	1.032(0.856-1.243)	0.743	2.549(2.204-2.949)	0.000	1.252(0.996-1.574)	0.054
Surgery Performed vs. others	2.169(1.964-2.395)	0.000	2.130(1.847-2.457)	0.000	3.244(2.876-3.658)	0.000	1.536(1.258-1.874)	0.000
Chemotherapy Yes vs. no	0.806(0.728-0.892)	0.000	1.042(0.934-1.163)	0.462	0.528(0.464-0.600)	0.000	1.032(0.891-1.194)	0.676
Radiotherapy Yes vs. no	0.700(0.632-0.775)	0.000	0.857(0.768-0.957)	0.006	0.376(0.333-0.425)	0.000	0.818(0.695-0.962)	0.015
Insurance Yes vs. no	0.996(0.831-1.194)	0.965			1.660(1.377-2.002)	0.000	1.611(1.317-1.972)	0.000
Bone mets					0.273(0.234-0.318)	0.000	0.729(0.603-0.881)	0.001
Brain mets					0.226(0.198-0.257)	0.000	0.524(0.437-0.628)	0.000
Liver mets					0.229(0.198-0.266)	0.000	0.539(0.446-0.651)	0.000
Lung mets					0.256(0.225-0.291)	0.000	0.667(0.562-0.792)	0.000

**Table 3 B:** Univariate and multivariate analysis of factors affecting the survival in non-ICI era and ICI in second age group.

Parameters	( 60-74 years) Non-ICI era				( 60-74 years) ICI era			
	Univariate HR (CI)	P value	Multivariate HR (CI)	P value	Univariate HR (CI)	P value	Multivariate HR (CI)	P-value
Sex Male vs. female	1.001(0.886-1.131)	0.988			0.791(0.694-0.901)	0.000	0.795(0.691-0.915)	0.001
Race								
White		0.217				0.948		
Black	0.808(0.548-1.192)	0.283			0.986(0.626-1.552)	0.951		
Others	0.771(0.537-1.109)	0.161			0.941(0.651-1.361)	0.746		
Marital	1.086(0.969-1.217)	0.155			1.265(1.128-1.418)	0.000	1.357(1.201-1.534)	0.000
Primary site								
Trunk		0.000		0.291		0.000		0.005
Upper site	0.848(0.680-1.057)	0.142	0.874(0.699-1.094)	0.240	0.907(0.735-1.118)	0.359	1.042(0.840-1.293)	0.709
Lower site	0.803(0.652-0.989)	0.039	0.828(0.669-1.024)	0.082	0.720(0.581-0.893)	0.003	0.825(0.657-1.036)	0.098
Others	1.321(1.140-1.531)	0.000	0.884(0.717-1.089)	0.247	1.634(1.414-1.888)	0.000	0.725(0.591-0.889)	0.002
Laterality								
Right		0.000		0.571		0.000		0.398
Left	1.092(0.920-1.297)	0.314	1.097(0.922-1.305)	0.296	0.919(0.778-1.085)	0.317	0.891(0.750-1.057)	0.185
Others	1.593(1.381-1.837)	0.000	1.066(0.855-1.330)	0.571	1.946(1.693-2.237)	0.000	0.970(0.784-1.200)	0.779
Surgery Performed vs. others	1.920(1.715-2.148)	0.000	1.877(1.610-2.189)	0.000	3.031(2.698-3.405)	0.000	2.103(1.750-2.527)	0.000
Chemotherapy Yes vs. no	0.960(0.850-1.083)	0.505			0.631(0.549-0.725)	0.000	1.062(0.909-1.240)	0.451
Radiotherapy Yes vs. no	0.750(0.665-0.845)	0.000	0.881(0.777-0.998)	0.047	0.476(0.422-0.538)	0.000	0.930(0.791-1.093)	0.379
Insurance Yes vs. no	1.069(0.791-1.445)	0.663			1.397(1.088-1.794)	0.009	1.236(0.943-1.620)	0.124
Bone mets					0.304(0.262-0.353)	0.000	0.596(0.500-0.711)	0.000
Brain mets					0.290(0.255-0.330)	0.000	0.524(0.439-0.625)	0.000
Liver mets					0.275(0.239-0.317)	0.000	0.596(0.500-0.709)	0.000
Lung mets					0.316(0.279-0.357)	0.000	0.645(0.550-0.756)	0.000

**Table 3 C.** Univariate and multivariate analysis of factors affecting the survival in non-ICI era and ICI in third age group.

Parameters	( >74years) Non-ICI era				( >74years) ICI era			
	Univariate HR (CI)	P value	Multivariate HR (CI)	P value	Univariate HR (CI)	P value	Multivariate HR (CI)	P-value
Sex Male vs. female	1.029(0.908-1.166)	0.651			0.830(0.722-0.955)	0.009	0.942(0.811-1.093)	0.482
Race								
White		0.209				0.616		
Black	0.751(0.487-1.158)	0.195			0.744(0.411-1.349)	0.331		
Others	0.771(0.510-1.166)	0.217			1.037(0.622-1.728)	0.890		
Marital	1.173(1.039-1.324)	0.010	1.140(1.007-1.291)	0.039	1.140(0.998-1.303)	0.054		
Primary site								
Trunk		0.000		0.148		0.000		0.464
Upper site	0.803(0.641-1.006)	0.056	0.859(0.681-1.083)	0.198	0.800(0.617-1.038)	0.093	0.946(0.721-1.243)	0.692
Lower site	0.780(0.628-0.968)	0.024	0.767(0.613-0.961)	0.021	0.744(0.574-0.964)	0.025	0.831(0.630-1.095)	0.188
Others	1.433(1.207-1.702)	0.000	0.908(0.727-1.133)	0.391	1.459(1.198-1.777)	0.000	0.859(0.674-1.094)	0.218
Laterality								
Right		0.000		0.749		0.000		0.734
Left	0.941(0.789-1.122)	0.496	0.960 (0.805-1.146)	0.653	0.914(0.750-1.115)	0.376	0.952(0.776-1.168)	0.637
Others	1.690(1.458-1.959)	0.000	1.050(0.837-1.316)	0.673	1.788(1.513-2.114)	0.000	0.905(0.704-1.164)	0.437
Surgery Performed vs. others	2.285(2.018-2.589)	0.000	2.089(1.774-2.459)	0.000	2.486(2.171-2.847)	0.000	1.642(1.335-2.019)	0.000
Radiation status Yes vs. no	0.756(0.653-0.877)	0.000	0.860(0.739-1.001)	0.051	0.644(0.550-0.754)	0.000	1.076(0.890-1.300)	0.450
Chemotherapy Yes vs. no	0.968(0.810-1.156)	0.718			0.727(0.595-0.890)	0.002	1.178(0.948-1.464)	0.140
Insurance Yes vs. no	1.185(0.821-1.710)	0.366			1.061(0.767-1.469)	0.719		
Bone mets					0.438(0.359-0.534)	0.000	0.829(0.655-1.048)	0.117
Brain mets					0.294(0.249-0.347)	0.000	0.412(0.334-0.509)	0.000
Liver mets					0.330(0.276-0.395)	0.000	0.514(0.416-0.637)	0.000
Lung mets					0.378(0.327-0.436)	0.000	0.633(0.529-0.759)	0.000

## Discussion

Age is an essential prognostic factor in patients with aggressive malignant melanoma, and the prognosis worsens with age (10). Differences in the natural history of melanoma among younger and older patients are speculated to be partly caused by immunosenescence, facilitating the escape of melanoma cells from effective immune surveillance (11). Clinical trials conducted in the last decade have investigated the effects of ICIs on various solid cancer types, including cancers that are difficult to treat, such as melanoma. Results have consistently revealed that ICIs can improve the OS of patients with malignant melanoma, either in combination with other ICI agents (i.e., ipilimumab and nivolumab) or in monotherapy (12, 13). However, systematic investigations on the influence of specific age on the prognosis of patients with melanoma receiving ICIs are largely missing.

In the present population-based study, the effects of ICIs on the different age groups were evaluated, and the differences among these groups in terms of survival were examined. The results revealed that ICIs had excellent survival benefits for younger patients than for elderly patients with melanoma. In the non-ICI era, there was no difference between the first (20-59) and second ( 60-74) age groups, with less favorable survival in the older group ( > 74). These results suggest that the benefits of immunotherapy have been introduced for better survival, but further age disparities have risen. The increase in survival was primarily attributed to the introduction of immunotherapeutic drugs in therapeutic regimens. This improvement was notably lower than that reported in prior studies (14), which designated only 24 months as the cutoff value for survival in the younger age group regardless of their status and other comorbidities. The present results also revealed that most variables demonstrated significant unfavorable OS in the age groups (i.e., first group) in the non-ICI era. Several studies have reported that ICI-based multimodal treatments can remarkably enhance the anticancer activity across different diseases (15). There have been reports on the effects of immunotherapy in older patients. While no difference in the survival among the non-younger groups after introducing ICI has been reported (16), another study has reported that a subgroup of older patients had greater survival than did the younger group (17). In addition to the cohort number and the different locations, these results could be related to the missed and non-included variables almost related to the impact of age. Another reason is that, assuming quality and high-performing healthcare settings, with universal healthcare, can more easily replicate the context of clinical trials (18, 19). However, this is insufficient to capture the full landscape of disparities in elderly care—and maybe oversimplified. Consequently, there are numerous variables that we cannot account for, resulting in the findings of our study.

In our study, the age groups in the ICI era were fairly treated with chemotherapy as its frequency is less than that of chemotherapy non-use. Thus, almost all ICIs were suggested to be administered in patients with melanoma, further supporting the present results of improved benefits in the ICI era compared to the non-ICI era. Selected patients with melanoma may be eligible for treatment with radiotherapy, brain disease control, or other palliative services (20). Our study showed a positive correlation between ICIs plus radiotherapy and OS, thereby supporting the idea of synergistic effects. However, without specific knowledge of the types and districts of delivery, this finding is largely speculative (21). The effect of radiotherapy seemed limited to the non-younger aged population; the results of Cox multivariate analysis confirmed that its effect was insignificant in non-younger patients. The patients in the ICI era who also underwent surgery had significantly better survival outcomes than those in the non-ICI era who did not undergo surgery, indicating that surgery could be a predictive factor for favorable survival in all age subgroups in the ICI era. In general, the combination of local–locoregional approaches to integrate and optimize ICI treatments in patients with melanoma is currently common and can help improve progression-free survival and or proceed beyond progression. This finding holds when the disease progression pattern during ICI treatment regards a few sites, which can be surgically approached or tackled with radiotherapy, resulting in one interpretation of the correlative analysis. Previous studies reported favorable survival when distant melanoma tissues were removed *via* surgery after checkpoint blockade, especially when they responded to ICI treatment (22, 23). Administration of chemotherapy was highly observed in patients in the non-ICI era, mostly because it was the only treatment modality available. Having an insurance policy played an important role in various treatment regimens, especially in the ICI era. Unlike younger age groups (20-59), the older ICI age groups OS was not related to insurance availability. Recent work has reported that melanoma patients younger than 65 years who were insured in the ICI era had higher OS than those who did not have an insurance policy (24, 25), suggesting an influence on the likelihood of accessing treatment affordably. At the same time, patients with mets had worse survival than did those in the non-mets group, and the difference in OS between the mets sites within each age group clearly increased throughout the ICI era. While brain metastasis was related to poor survival in the older group, the liver mets site was poorly associated with shorter survival in most cases and coincided with the results of a cohort of clinical trials (26).

The present study has several limitations. Firstly, the database we consulted did not expound on specific age groups with melanoma. Secondly, the short median OS was associated with a 24-month follow-up only, comorbidities were not examined, and there was lack of information on the performance status. There was no chance to clarify whether patients had received anti-CTLA4, anti-PD1, or both. Eventually, we could not investigate whether elderly patients had experienced more toxicity from ICIs, impairing the dose exposure and reducing the durable benefit of disease control, and what comorbidities were specifically prominent, including the treatments received and the need for corticosteroids or antibiotics for other diseases.

## Conclusion

Our results indicated that age-related disparities might affect the OS outcomes in patients with metastatic melanoma in both the ICI and non-ICI eras. ICIs had clear significant effects on all groups, but significantly improved the OS of patients younger than 59 years. Surgical removal of metastatic melanoma and having an insurance policy were found to be positive predictors for OS in most cases. In addition to the independent predictive factor of surgery, this study highlights the importance of access to government-sponsored insurance programs in overcoming age-based inequalities in healthcare outcomes, especially in the era of ICI.

## Data Availability Statement

The original contributions presented in the study are included in the article/**Supplementary Material**. Further inquiries can be directed to the corresponding author.

## Author Contributions

MS and JL conceptualized the study. MS, MA, and CJ contributed to validation. MS, MA, and CJ did the formal analysis. MS, MA, CJ contributed to the investigation and prepared the original draft. DT, SA, AW, SB, BA, HA, and XS contributed to visualization. JL supervised the study. MS and JL administered the project. All authors contributed to the article and approved the submitted version.

## Funding

This research was funded by the National Natural Science Foundation of China, Grant/Award Numbers: 8217101453.

## Conflict of Interest

The authors declare that the research was conducted in the absence of any commercial or financial relationships that could be construed as a potential conflict of interest.

## Publisher’s Note

All claims expressed in this article are solely those of the authors and do not necessarily represent those of their affiliated organizations, or those of the publisher, the editors and the reviewers. Any product that may be evaluated in this article, or claim that may be made by its manufacturer, is not guaranteed or endorsed by the publisher.

## Supplementary Material

Supplementary File 1 KM OS difference in age groups of non-ICI era vs. ICI era.

Supplementary File 2 KM OS differences of metastatic sites in each age groups of ICI era.
