# Supplementary figures and images for: Age-Based Disparities in Metastatic Melanoma Patients Treated in the Immune Checkpoint Inhibitors (ICI) Versus Non-ICI Era: A Population-Based Study
Source: Front Immunol. 2021 Nov 16;12:609728. doi: 10.3389/fimmu.2021.609728 (PMC8650702; doi:10.3389/fimmu.2021.609728)

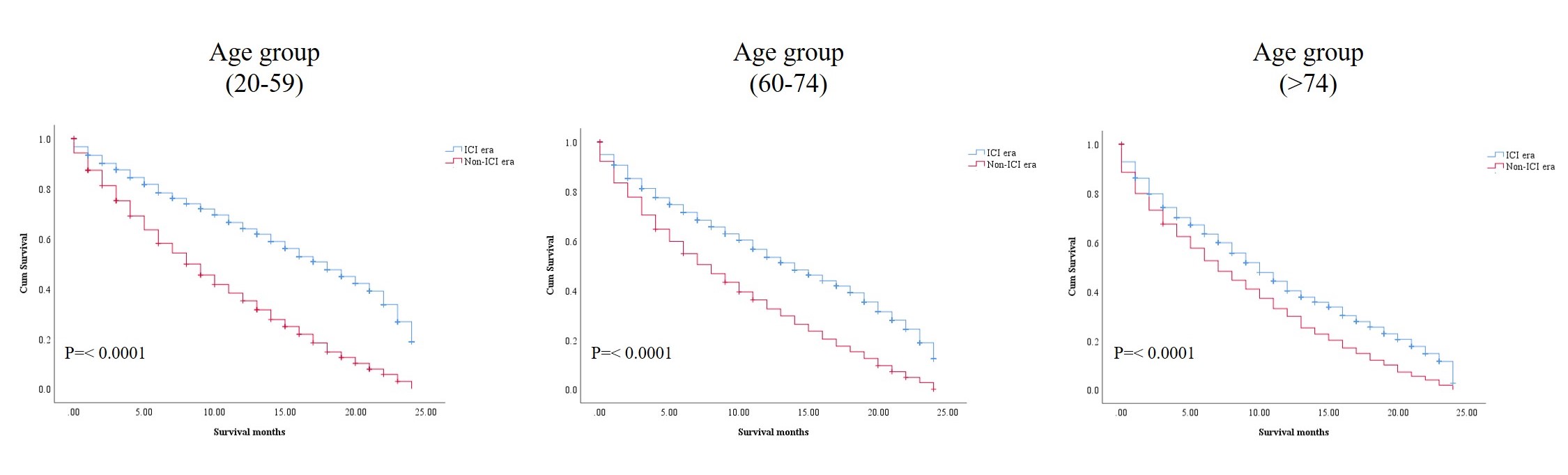

Supplement: Supplementary File 1 — KM OS difference in age groups of non-ICI era vs. ICI era. [file Image_1.tif]

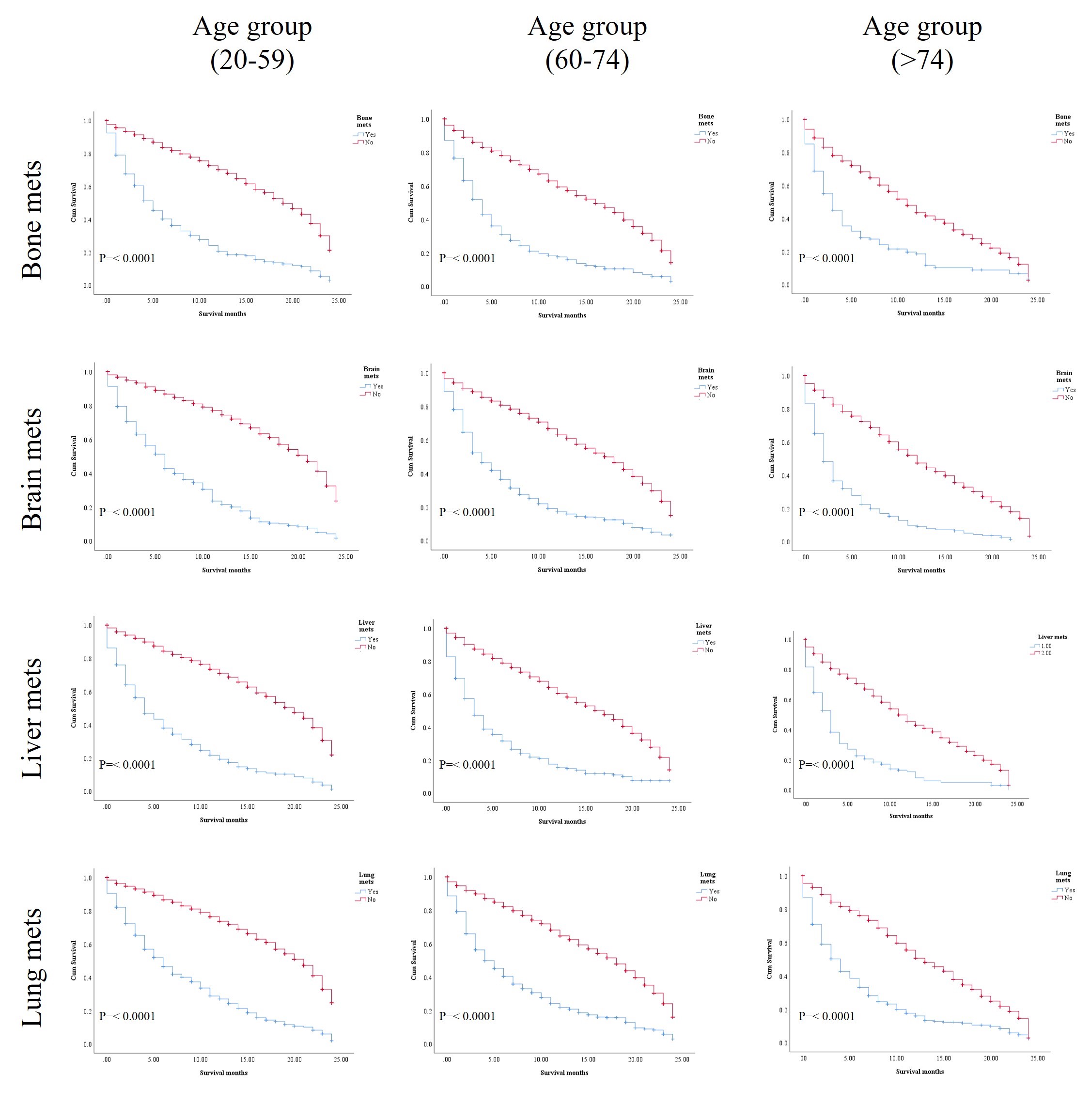

Supplement: Supplementary File 2 — KM OS differences of metastatic sites in each age groups of ICI era. [file Image_2.tif]
